# Second-Hand Smoke Increases Bronchial Hyperreactivity and Eosinophilia in a Murine Model of Allergic Aspergillosis

**DOI:** 10.1080/10446670310001598483

**Published:** 2003-03

**Authors:** Brian W. P. Seymour, Edward S. Schelegle, Kent E. Pinkerton, Kathleen E. Friebertshauser, Janice L. Peake, Viswanath P. Kurup, Robert L. Coffman, Laurel J. Gershwin

**Affiliations:** 1 Department of Pathology, Microbiology and Immunology 1126 Haring Hall School of Veterinary Medicine, University of California One Shields Avenue Davis CA 95616 USA; 2 From the Department of Immunobiology DNAX Research Institute of Molecular and Cellular Biology 901 California Avenue Palo Alto CA 94304 USA; 3 Department of Anatomy Physiology and Cell Biology School of Veterinary Medicine University of California Davis CA 95616 USA; 4 Department of Medicine Allergy Immunology Division Medical College of Wisconsin Research Service 151-I, V.A. Medical Center Milwaukee WI 53295-1000 USA; 5 Dynavax Technologies 717 Poller St., Suite 100 Berkeley CA 94710 USA

## Abstract

Involuntary inhalation of tobacco smoke has been shown to aggravate the allergic response. Antibodies to fungal antigens such as *Aspergillus fumigatus* (Af) cause an allergic lung disease in humans. This study was carried out to determine the effect of environmental tobacco smoke (ETS) on a murine model of allergic bronchopulmonary aspergillosis (ABPA). BALB/c mice were exposed to aged and diluted sidestream cigarette smoke to simulate 'second-hand smoke'. The concentration was consistent with that achieved in enclosed public areas or households where multiple people smoke. During exposure, mice were sensitized to Af antigen intranasally. Mice that were sensitized to Af antigen and exposed to ETS developed significantly greater airway hyperreactivity than did mice similarly sensitized to Af but housed in ambient air. The effective concentration of aerosolized acetylcholine needed to double pulmonary flow resistance was significantly lower in Af + ETS mice compared to the Af + AIR mice. Immunological data that supports this exacerbation of airway hyperresponsiveness being mediated by an enhanced type 1 hypersensitivity response include: eosinophilia in peripheral blood and lung sections. All Af sensitized mice produced elevated levels of IL4, IL5 and IL10 but no IFN-γ indicating a polarized Th2 response. Thus, ETS can cause exacerbation of asthma in ABPA as demonstrated by functional airway hyperresponsiveness and elevated levels of blood eosinophilia.


*Clinical & Developmental Immunology*, March 2003, Vol. 10 (1), pp. 35–42

Taylor & Francis healthsciences

### Second-Hand Smoke Increases Bronchial Hyperreactivity and Eosinophilia in a Murine Model of Allergic Aspergillosis

BRIAN W.P. SEYMOUR[a,b], EDWARD S. SCHELEGLE[c], KENT E. PINKERTON[c], KATHLEEN E. FRIEBERTSHAUSER[a], JANICE L. PEAKE[c], VISWANATH P. KURUP[d], ROBERT L. COFFMAN[e] and LAUREL J. GERSHWIN[a,*]

[a]*Department of Pathology, Microbiology and Immunology, 1126 Haring Hall, School of Veterinary Medicine, University of California, One Shields Avenue, Davis, CA 95616, USA;* [b]*From the Department of Immunobiology, DNAX Research Institute of Molecular and Cellular Biology, 901 California Avenue, Palo Alto, CA 94304, USA;* [c]*Department of Anatomy, Physiology and Cell Biology, School of Veterinary Medicine, University of California, Davis, CA 95616, USA;* [d]*Department of Medicine, Allergy Immunology Division, Medical College of Wisconsin, Research Service 151-I, V.A. Medical Center, Milwaukee, WI 53295-1000, USA;* [e]*Dynavax Technologies, 717 Poller St., Suite 100, Berkeley, CA 94710, USA*

Involuntary inhalation of tobacco smoke has been shown to aggravate the allergic response. Antibodies to fungal antigens such as *Aspergillus fumigatus* (Af) cause an allergic lung disease in humans. This study was carried out to determine the effect of environmental tobacco smoke (ETS) on a murine model of allergic bronchopulmonary aspergillosis (ABPA). BALB/c mice were exposed to aged and diluted sidestream cigarette smoke to simulate ‘second-hand smoke’. The concentration was consistent with that achieved in enclosed public areas or households where multiple people smoke. During exposure, mice were sensitized to Af antigen intranasally. Mice that were sensitized to Af antigen and exposed to ETS developed significantly greater airway hyperreactivity than did mice similarly sensitized to Af but housed in ambient air. The effective concentration of aerosolized acetylcholine needed to double pulmonary flow resistance was significantly lower in Af + ETS mice compared to the Af + AIR mice. Immunological data that supports this exacerbation of airway hyperresponsiveness being mediated by an enhanced type 1 hypersensitivity response include: eosinophilia in peripheral blood and lung sections. All Af sensitized mice produced elevated levels of IL4, IL5 and IL10 but no IFN-γ indicating a polarized Th2 response. Thus, ETS can cause exacerbation of asthma in ABPA as demonstrated by functional airway hyperresponsiveness and elevated levels of blood eosinophilia.

*Keywords*: Environmental tobacco smoke; Mice; Cytokine; Immunoglobulin; Allergy

### INTRODUCTION

The inhalation of environmental tobacco smoke (ETS) also known as “second-hand smoke” has been shown to have adverse effects on the health of nonsmokers. Epidemiological studies have concluded that exposure to ETS is responsible for some forms of cancer, respiratory disease and may also exacerbate the allergic response (Martinez *et al.*, 1988; Stone, 1992; El-Nawawy *et al.*, 1996; Gilliland *et al.*, 2001). Studies on human subjects are rarely able to focus on ETS alone due to concurrent exposure to other environmental pollutants (such as ozone, diesel exhaust) which may also modulate the immune response. For this reason, we have previously studied the effects of ovalbumin (OVA) -sensitized mice in aged and diluted side stream cigarette smoke (Seymour *et al.* 1997; Seymour *et al.*, 2002) produced by a smoke generation system that simulates “second-hand smoke” (Teague *et al.*, 1994). In this model, we found that OVA-sensitized BALB/c mice exposed to ETS resulted in the upregulation of the allergic response. There were increases in the cellularity of the bronchoalveolar lavage (BAL) and in T helper cell type 2 (Th2) cytokines of the lungs, particularly IL4 and IL10. Serum and blood analysis revealed increased levels of circulating IgE, IgG1 and eosinophils from ETS exposed OVA-sensitized mice. The effects of ETS were more profound on females when compared to male mice (Seymour *et al.*, 2002). We (Seymour *et al.*, 1998) and others (McMenamin *et al.*, 1994) have shown that repeated exposure of naive adult mice to aerosolized OVA resulted in IgE unresponsiveness. However Rumold *et al.* (2001) have demonstrated that exposure of naïve adult mice to aerosolized OVA along with ETS resulted in elevated levels of serum IgE and eosinophils in the BAL. These observations prompted an interest in further investigating the effects of ETS on exposure to a clinically relevant and more complex antigen. Thus, we chose to examine the effects of ETS on an established animal model of allergic bronchopulmonary aspergillosis (ABPA) (Kurup *et al.*, 1992).

*Corresponding author. Tel.: +1-530-752-6643. Fax: +1-530-752-3349. E-mail: ljgershwin@ucdavis.edu

ISSN 1740-2522 print/ISSN 1740-2530 online © 2003 Taylor & Francis Ltd
DOI: 10.1080/1044667031000159848

ABPA is a complex hypersensitivity lung disease caused by the inhalation of *Aspergillus fumigatus* (Af) antigens (reviewed in Grunig *et al.* (1998a)), with components of both immediate and delayed type hypersensitivity. This opportunistic fungal pathogen is generally found in homes particularly in the farming population (Kauffman *et al.*, 1995). ABPA is prevalent among patients with cystic fibrosis (Laufer *et al.*, 1984) and is characterized by elevated levels of serum IgE and eosinophils indicating a Th2 response (Kauffman *et al.*, 1995). Indeed, in a murine model of ABPA, Th2 cytokines, particularly IL4 and IL5, were detected in cell cultures from the lungs (Kurup *et al.*, 1992; Kurup *et al.*, 1994a) and BAL (Grunig *et al.*, 1997) of Af sensitized mice and administration of antibodies to IL4 and IL5 abrogated the IgE and eosinophilia in these animals (Kurup *et al.*, 1994b).

Enhanced allergic sensitization to Af and other fungal antigens, as demonstrated by skin tests, has been reported in atopic children (Ronchetti *et al.*, 1992). However, investigators have also reported that the incidence of ABPA is less prevalent in smokers than in nonsmokers (reviewed in Holt (1987)). Studies have revealed that the allergic response from Af antigen is an event involving not only Th2 but also the Th1 cytokine, IFN-$\gamma$ that is regulated by endogenous IL10 (Grunig *et al.*, 1997). Thus ABPA has a more complex pathogenesis than simple type 1 hypersensitivity as demonstrated in the OVA model of allergy (Seymour *et al.*, 1997). Although the mechanism through which cigarette smoke modifies risk factors to ABPA is unknown, we believe it is immunologic in nature. In order to understand how cigarette smoke may influence this disease, we exposed BALB/c mice to ETS, while concurrently delivering via the intranasal (i.n.) route, Af or phosphate buffered saline (PBS). We hypothesized that development of airway hyperresponsiveness, eosinophilia and production of Th2 cytokines would be enhanced by exposure to ETS.

### METHODS

### Animals

Specific pathogen-free BALB/c mice from Charles Rivers (Hollister, CA) were 8–9 weeks old at the start of each experiment. Female mice were used to avoid injury and subsequent inflammation that often results from male fighting.

### Preparation and Administration of the Af Antigen

The Af antigen was a mixture of mycelial extract and culture filtrate. Af was grown and antigen prepared as previously described (Kurup *et al.*, 1992). Briefly, Af was grown in synthetic broth for 2–3 weeks. The culture filtrate was dialyzed against deionized water and freeze-dried. A French press (S.L.M. Instruments, Urbana, IL) was used for extraction of the mycelium extract. This was done by homogenizing the mycelium at 10,000 psi for extraction of the antigen. These antigens were evaluated for their protein content and their level of reactivity with patients' serum. Two hundred micrograms of Af was evaluated to have 50 $\mu$g of protein. These exposures were done by using a pipette to instill the Af i.n. into mice anesthetized lightly with ether.

### Experimental Protocol

BALB/c mice were exposed to ETS or ambient air from day 0 through day 43 (Fig. 1). They were sensitized by instillation of 50 $\mu$l of soluble Af antigen into their nostrils on days 14, 17, 21 and 24. In three separate experiments different doses of Af were studied: 200, 100, 50 and 25 $\mu$g. Control mice were given 50 $\mu$l of PBS i.n. and exposed either to ETS or ambient air from days 0 through 43. The ETS concentration was at a high ambient level, but similar to those observed in restaurants, bars and the homes of smokers. During ETS/ambient air exposures animals were bled from their tail veins for IgE antibodies

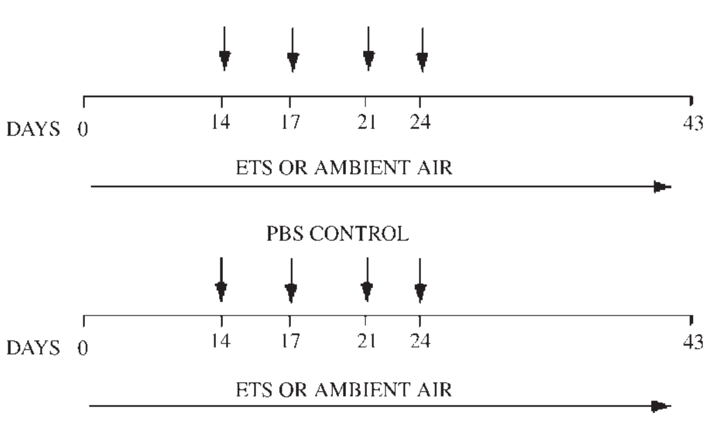

FIGURE 1 Antigen sensitization and ETS/AIR exposure protocols.

and eosinophil determination. Cytokine production was carried out by *in vitro* stimulation of lung cells on day 4 after the final Af or PBS challenges.

### Research Cigarettes

The cigarette used in this study was the IR4F that is a filtered cigarette used by research laboratories. They were obtained from the Tobacco and Health Research Institute (University of Kentucky, Lexington, KY). Once purchased, they were stored at 4°C until used in an experiment. Two days before use, they were placed at 23°C in a chamber containing water and glycerol (this mixture was at a ratio of 0.76:0.26) in order to achieve a relative humidity of 60%.

### The Smoke Generation System

The smoke generation system was designed by Teague and colleagues (Teague *et al.*, 1994). Whenever the animals were not receiving ETS, a source of filtered air was delivered to the mice so that mice exposed to ETS can be housed in these chambers for the duration of the experiment.

### ETS Exposure

Mice were exposed to ETS for 6 h per day from Monday through Friday. At the end of the daily exposure, the smoke generator was turned off but the animals remained in the exposure chambers. Each exposure chamber had a measurement of $69 \times 69 \times 61\,cm^3$ with a complete change of air every 3 min. Up to 48 mice in 12 cages were housed in a single chamber.

The total suspended particulates (TSP) from the experiment in which mice were exposed to 200 μg Af per exposure revealed a TSP concentration of $1.00 \pm 0.15\,mg/m^3$. The nicotine and carbon monoxide concentrations were $159 \pm 55\,\mu g/m^3$ and $6.6 \pm 0.9$ ppm, respectively. The relative humidity was $41.0 \pm 9.0\%$ while the temperature was $23.0 \pm 1.0$°C. The TSP from the experiment in which mice were challenged with low doses of Af (25, 50 and 100 μg Af per exposure), was $1.00 \pm 0.05\,mg/m^3$. The nicotine and carbon monoxide concentrations were $237 \pm 109\,\mu g/m^3$ and $6.8 \pm 0.9$ ppm, respectively. The relative humidity was $48.0 \pm 10\%$ while the temperature was $20 \pm 1.0$°C. Measurements of TSP and nicotine after the system was turned off revealed a rapid decline to nondetectable levels ($< 15\,\mu g/m^3$) during the nonsmoking period.

### Airway Responsiveness Measurements

Airway responsiveness to increasing concentrations of acetylcholine aerosols was determined in 110 mice. The mice studied were divided into eight experimental groups, that included: ambient air exposed with nasal instillation of PBS ($n = 16$); ambient air exposed with nasal instillation of 25 μg Af/exposure ($n = 12$); ambient air exposed with nasal instillation of 50 μg Af/exposure ($n = 11$) ambient air exposed with nasal instillation of 200 μg Af/exposure ($n = 11$); ETS exposed with nasal instillation of PBS ($n = 23$); ETS exposed with nasal instillation of 25 μg Af per exposure ($n = 12$); ETS exposed with nasal instillation of 50 μg Af per exposure ($n = 13$); ETS exposed with nasal instillation of 200 μg Af per exposure ($n = 12$).

On the day of pulmonary function testing the mice were weighed, anaesthetized with an i.p. injection of 2.5% α-chloralose and 25% urethane (0.006 ml/gr body weight) and their trachea were cannulated. Next, the mice were placed in a whole body plethysmogaph and their tracheal cannula attached to a constant volume positive pressure ventilator (model 683, Harvard Inc.) set at a tidal volume of 0.20 ml and a frequency of 120 breaths/min. The mice were then paralyzed with succinyl chlorine (0.05 ml of 20 mg/ml, i.p.) and the plethysmograph was closed. Air flow was measured using a pneumotachograph (model 8300, Hans Rudolph Inc.) mounted into the lid of the plethysmograph. Airway pressure was measured using a differential pressure transducer (Statham Laboratories, Inc.) with one side attached near the tracheal cannula port of the ventilator and the other side attached to a port on the side of the plethysmograph. The air flow and airway pressure signals were amplified and then recorded and analyzed using a digital data acquisition system (PO-NE-MAH, Inc.). Pulmonary flow resistance ($R_L$) was calculated according to the method of Amdur and Mead (Amdur and Mead, 1958) with air flow and airway pressure being measured at an isovolume of 70% of tidal volume. Doubling concentrations of acetylcholine solutions, starting at 0.125 mg/ml were aerosolized using a jet nebulizer (Miniheart, Vortran, Inc.) and delivered to the mice by passing the aerosol stream past the inlet of the ventilator. Increasing concentrations of acetylcholine were used until $R_L$ doubled or a concentration of 64 mg/ml acetylcholine was reached. $R_L$ was corrected for the known resistance of each tracheal cannula. The effective concentration needed to double $R_L$ (EC200$R_L$) was calculated by interpolation of the log/log plot of the acetylcholine dose response curve.

### Quantification of Eosinophils in the Peripheral Blood

Eosinophils were quantified by dilution of heparinized blood in Discombe's fluid as previously described (Colley, 1972). This cell suspension was placed on a hemocytometer and eosinophils counted with a light microscope.

### Examination of Eosinophils in Lung Tissue Sections

To determine the presence of eosinophils in lung tissues, 5 μm thick paraffin sections were stained with hematoxylin and eosin and with a special stain for eosinophils and mast cells (combined Eosinophil/Mast cell stain [CEM], American Master Tech Scientific, Lodi, CA).

### *In-vitro* Stimulation of Homogenized Lung Cells

Mice were euthanized and their lungs removed aseptically, cut into small pieces and forced against a sterile No. 100 steel mesh (Tylinter Inc., Mentor, OH) with the piston of a 10 ml plastic syringe. The cells were washed three times in Hank's solution (GIBCOBRL, Grand Island, NY) then suspended in culture medium at $5 \times 10^6$/ml. The culture medium consisted of RPMI 1640 (J.H.R. Bioscience, Lenexa, KS.) with 10% heat-inactivated fetal calf serum (FCS) (Sigma, St Louis, MO.), 0.05 mM 2-ME (Sigma, St Louis, MO.), 2 mM L-glutamine (J.H.R. Bioscience, Lenexa, KS.) and penicillin/Streptomycin (GIBCO BRL, Life Technologies Inc., Grand Island, NY). Flat-bottom 24-well plates (Falcon; Becton Dickinson Labware, Lincoln Park, NJ) containing 1 ml of 20 μg/ml of hamster anti-mouse CD3 antibody (clone 145-2C11) were incubated overnight at 5°C. The following day, the solution was removed and wells were washed by adding 1 ml of cold PBS. The PBS was removed and the wash step repeated twice. One milliliter of each cell suspension was added to each well. The supernatant was harvested at 72 h.

### Cytokine ELISA

Sandwich ELISAs were done to measure IL5, IL10 and IFN-γ as previously described (Abrams, 1995). Briefly, ELISA plates were coated with the appropriate anti-cytokine antibodies and incubated at 4°C overnight. After incubation, plates were blocked for 30 min at room temperature by adding 150 μl of 20 % FCS in PBS containing 0.04% TWEEN 20 (Sigma, St Louis MO.) (FCS-PBST) to each well. Samples were diluted in 5% FCS-RPMI and added to each well at a volume of 50 μl per well. Plates were incubated overnight at 4°C then washed and the second-step antibody that was diluted in FCS-PBST was added at 50 μl per well. Plates were incubated for 1 h then washed and the enzyme conjugate that was diluted in FCS-PBST was added to each well. Plates remained at room temperature for 1 h after which they were washed and 100 μl per well of substrate containing 1 mg/ml 2,2′-azinobis(3-ethylbenzthiazoline-sulfonic acid) (Sigma) and 0.003% $H_2O_2$ in 0.1 M $Na_2HPO_4$ and 0.05 M citric acid was added. After the substrate was developed, the reaction was stopped by applying 0.05 ml of 0.2 M citric acid solution to each well. The plates were read on an ELISA reader (Molecular Devices Corporation, Menlo Park, CA).

### Analysis of IL4

IL-4 from cultured supernatant was detected by a bioassay using the IL4 dependent CT.4S cell line (kindly donated by Dr William Paul, NIH) as previously described (Seymour *et al.*, 1997). Briefly, CT.4S cells were resuspended in culture medium at a concentration of $1 \times 10^5$/ml of medium. Standard and supernatants to be tested were titered onto 96-well flat bottom plates at 50 μl/well. The range of the standard was 100 to 0.78 pg/ml. The culture medium containing the CT.4S cells was added to the wells at a volume of 50 μl/well. Plates were incubated for 72 h at 37°C in a humidified atmosphere containing 5% $CO_2$. At the end of the incubation period, 10 μl of 3-(4,5-dimethylthiazol-2-yl)-2,5-diphenyl tetrazolium bromide (Sigma) was added to each well followed by 150 μl of 0.04N HCL in isopropanol and the absorbance measured by an ELISA reader.

### Passive Cutaneous Anaphylaxis (PCA)

Pooled serum samples from each group were serially diluted in sterile PBS solution. Two white rats (250–300 gm) were anesthesized with ether and their backs were shaved. Intradermal injections were made (0.1 ml) of each dilution. Negative control consisted on PBS. Separate aliquots of serum were heat inactivated at 56°C for 30 min, as a control for non-IgE cytophilic antibodies (Braga and Mota, 1976). Twenty four hours after sensitization rats were injected IV with 5 mg OVA and 5 mg Evan's blue dye in PBSS. After 20 min blue circles on the skin were observed and measured.

### Statistics

Levels of antibodies, eosinophils and cytokines were calculated as mean and standard error of the mean. The two-tailed $p$ values were calculated according to the Mann-Whitney Test. A value of $p < 0.05$ was considered significant. These statistical procedures permitted comparison between PBS control groups and the corresponding various doses of Af antigen groups as well as between air and ETS groups. Significance was considered at a $p$-value of $<0.05$. The airway responsiveness data was expressed as the log of the EC200$R_L$ and analyzed using analysis of variance with Scheffe's joint estimation was used for post hoc analysis (Statview, Abacus Concepts, Inc.). A value of $p < 0.05$ was considered significant.

### RESULTS

### Increase in Airway Responsiveness in Af Sensitized and PBS Control Mice Exposed to ETS

To determine if ETS without exposure to Af has an effect on airway responsiveness, we compared EC200$R_L$ in the PBS + ETS group to the PBS + AIR group. Significant reduction in EC200$R_L$ was observed in the PBS + ETS group when compared to the PBS + AIR group (8.368 ± 1.457 mg/ml vs. 24.332 ± 5.735 mg/ml; $p < 0.05$) (Fig. 2). Mice that had either 50 or 200 μg of Af per exposure had depressed lung function. Because of their fragile state, a large number of the mice in these groups were lost during the process of performing airway responsiveness measurements. As a result of the bias that the loss of the most severely affected mice in these groups had on our analysis, data from the 200 ug Af groups was not

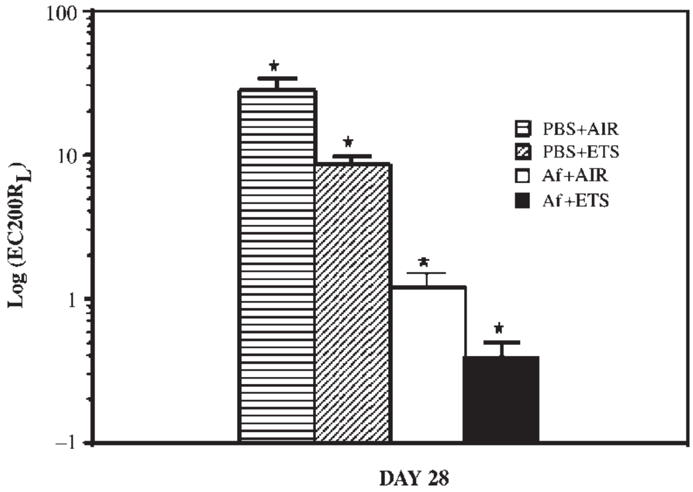

FIGURE 2 The effect of Af and ETS on airway responsiveness to acetylcholine aerosol in BALB/c mice. Animals were given 25 μg Af per exposure or PBS as described in Fig. 1 and airway responsiveness was measured on day 4 after the final i.n. sensitization. Airway responsiveness is expressed as the log of the effective concentration of acetylcholine that doubles pulmonary flow resistance (Log ($EC200R_L$)). *The mean $EC200R_L$ for each group was significantly different ($p < 0.05$) from each other group.

included in the final analysis; data reported is from only the mice that had 25 μg Af per exposure. Analysis of the 25 μg Af + AIR and Af + ETS groups showed significant differences in $EC200R_L$ between these groups ($1.208 \pm 0.301$ mg/ml vs. $0.394 \pm 0.105$ mg/ml, respectively $p < 0.05$).

### ETS Increases Blood Eosinophilia in Af Sensitized and PBS Control Mice

Previous studies have shown that i.n. administration of Af antigen results in increased numbers of eosinophils in the blood for 5–7 days after sensitization followed by a decline to baseline in about 2 weeks (Kurup *et al.*, 1994a). In the present study there was an increase in blood eosinophils of all mice exposed to the 200 ug Af antigen and also the 100 ug antigen dose (Fig. 3). At peak response, 3 and 5 days (Fig. 3) after the final i.n. antigen exposure (day 27 and 29, respectively), eosinophils were significantly higher in the Af + ETS group when compared to the Af + AIR group. By day 20 after the final i.n. sensitization, eosinophils had declined in all Af exposed groups but remained elevated above the PBS + AIR group. In another experiment, we observed that at this timepoint, eosinophils in the PBS + ETS group were significantly elevated to $59.2 \pm 8.6 \times 10^4$ compared to $28.0 \pm 3.8 \times 10^4$ in the PBS + AIR group ($p = 0.0089$; $n = 10$). Significant enhancement of eosinophil numbers

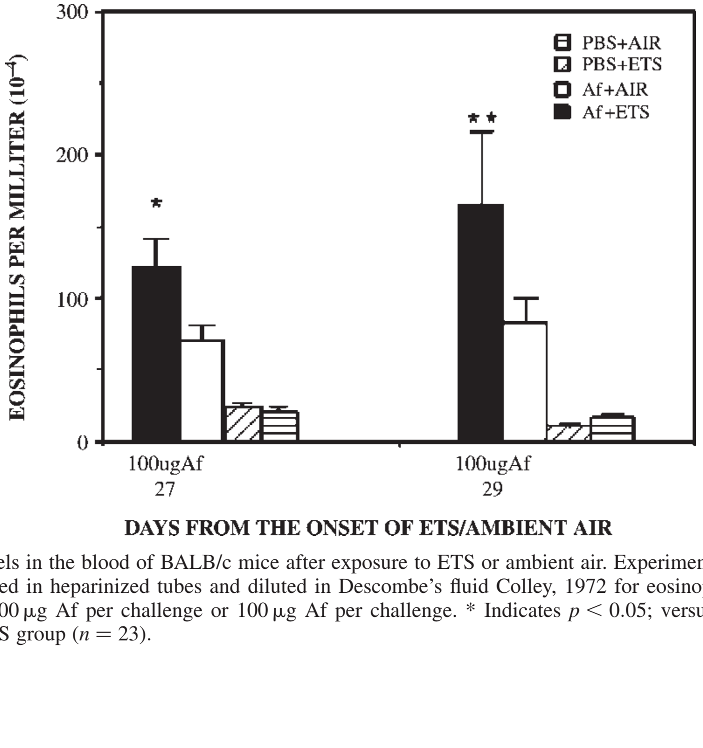

FIGURE 3 Eosinophil levels in the blood of BALB/c mice after exposure to ETS or ambient air. Experimental protocol was as described in Fig. 1. Blood samples were collected in heparinized tubes and diluted in Descombe's fluid Colley, 1972 for eosinophil determination. BALB/c mice were sensitized i.n. with either 200 μg Af per challenge or 100 μg Af per challenge. * Indicates $p < 0.05$; versus all other groups ($n = 10$). †Indicates $p = 0.0177$ versus Af + ETS group ($n = 23$).

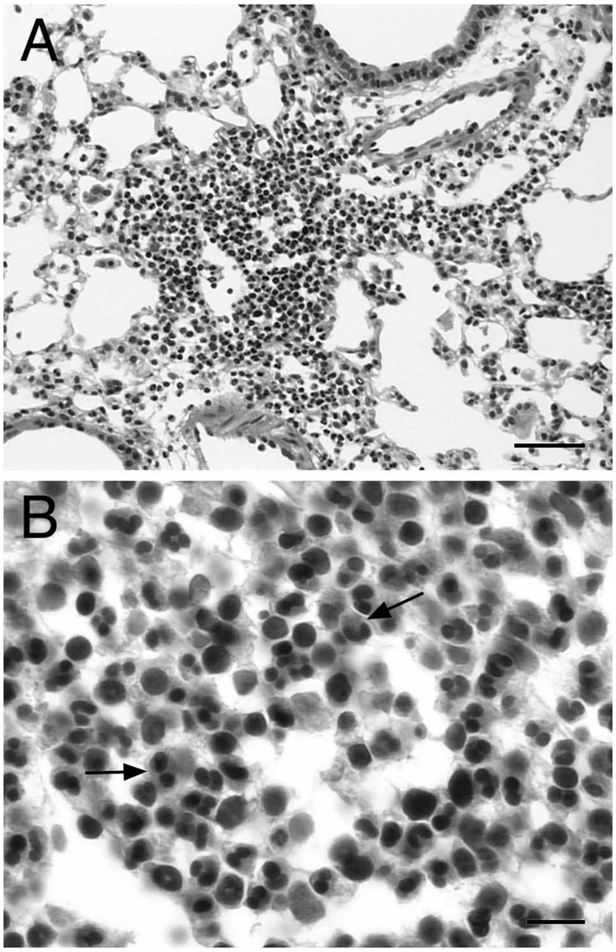

FIGURE 4 H and E stained paraffin section from the Af (high dose) +AIR group. Area of perivascular inflammation found in both air and ETS groups (panel A, bar = 50 μm). The perivascular influx contains eosinophils (arrow), (panel B, bar = 10 μm).

in the PBS + ETS group was generally seen after 6 weeks of ETS exposure. Peripheral eosinophilia was not seen with the 25 ug dose.

### Demonstration of Eosinophil Infiltration in Lungs of Mice Exposed to Af

In lung sections from both Af + AIR and Af + ETS exposed mice, areas of perivascular accumulation of inflammatory cells contained numerous eosinophils as well as some lymphocytes (Fig. 4). The eosinophilic cell infiltrate appeared to be similar in both AIR and ETS exposed groups, but was not present in lung sections that were not sensitized with Af.

### Aspergillus Specific IgE Measured by PCA

Pooled serum from mice receiving the 100 ug dose of Af antigen in the ETS environment showed PCA titers greater than 16. Similarly mice receiving Af antigen in filtered air had PCA titers greater than 16. In contrast mice exposed only to PBS in air or ETS showed negative PCA tests. Heat inactivated samples were also negative.

### Cytokine Production by Lung Cells Stimulated with Anti-CD3

Homogenized lungs from all groups were stimulated *in vitro* with plate-bound anti-CD3 on day 4 after the last i.n. challenge and cytokine profiles were evaluated. The PBS + AIR group made detectable levels of IL5, IL10 and IFN-γ but not IL4 (Table I). IL4, IL5 and IL10 were significantly ($p < 0.005$ for IL5 and IL10) elevated in both Af sensitized groups when compared to the PBS control groups, while IFN-γ was not detectable. This pattern is demonstrative of a polarized Th2 response. However, there were no significant differences in the cytokine responses from the homogenized lung of Af + ETS group when compared to the Af + AIR group.

### DISCUSSION

The experiments described herein focused on the effect of ETS on BALB/c mice sensitized by the intranasal route to Af antigen. The immunomodulatory effect of tobacco smoke and its components have been well documented (Thomas *et al.*, 1975; Ronchetti *et al.*, 1990; Stone, 1992; Sopori *et al.*, 1993; Abrams, 1995; Geng *et al.*, 1995; El-Nawawy *et al.*, 1996; Geng *et al.*, 1996; Oryszczyn *et al.*, 2000; Gilliland *et al.*, 2001). In our previous studies we showed that prolonged exposure of OVA sensitized mice to ETS resulted in enhanced levels of serum IgE, IgG1, and blood eosinophils (Seymour *et al.*, 1997). Experimental ABPA has been well described in the mouse model (Grunig *et al.*, 1998a). Mice exposed by the i.n. route to Af antigen characteristically develop high total IgE, IgG1, peripheral and pulmonary eosinophilia and

TABLE I Cytokines produced after anti-CD3 stimulation of homogenized lung cells from Af sensitised and control mice

| Sensitization (i.n.) | Exposure | IL4 ng/ml | IL5 ng/ml | IL10 u/ml | IFN-γ ng/ml |
|---|---|---|---|---|---|
| PBS | AIR | <0.08 | 0.99 ± 0.31 | 6.3 ± 1.0 | 3.45 ± 0.96 |
| PBS | ETS | <0.08 | 0.50 ± 0.05 | <2.5 | 3.65 ± 0.89 |
| 100 μg Af/challenge | AIR | 0.25 ± 0.06 | 2.70 ± 0.80 | 13.6 ± 6.4 | <0.313 |
| 100 μg Af/challenge | ETS | 0.44 ± 0.16 | 2.00 ± 0.40 | 11.0 ± 3.7 | <0.313 |
| 200 μg Af/challenge | AIR | 0.35 ± 0.05 | 3.24 ± 0.36 | 21.6 ± 2.5 | <0.313 |
| 200 μg Af/challenge | ETS | 0.57 ± 0.11 | 3.66 ± 0.41 | 19.1 ± 1.8 | <0.313 |

*In vitro* stimulation was performed on day 4 after final i.n. sensitization as described in the methods section. Results show the mean and SEM of 12 mice per group.

bronchial hyperresponsiveness (Wang *et al.*, 1994). Herein, we reported that Af sensitized mice developed high peripheral and pulmonary eosinophilia, bronchial hyperresponsiveness and allergen-specific IgE as expected. However, when ETS exposed Af sensitized mice were compared to filtered air housed Af sensitized mice, our data showed statistically significant ETS exacerbation of airway hyper-responsiveness and peripheral eosinophilia.

A significant elevation of eosinophils was observed in the blood of PBS + ETS mice after 6 weeks of ETS exposure demonstrating that prolonged exposure to ETS alone can enhance eosinophilia in mice. Similarly, studies in humans have also shown significant blood eosinophilia, independent of serum IgE, as a result of inhalation of second-hand smoke (Ronchetti *et al.*, 1990). Tobacco smoke, a complex mixture of over 4000 different chemicals (Stedman, 1968) contains substances that can induce inflammation and respiratory irritation (Shephard, 1992; Witschi *et al.*, 1997). Indeed, these substances have been shown to activate histamine from mast cells in smokers (reviewed in Holt, 1987). The fragments from the mycelia of Af, a component of the Af extract used in this study, can induce degranulation of sensitized mast cells to secrete chemotactic factors as well as Th2 type cytokines (Kauffman *et al.*, 1995). Moreover, mast cell degranulation by IgE antibody results in both bronchoconstriction and eosinophil chemotaxis (Wardlaw *et al.*, 1988). We examined pooled sera from the Af + ETS and pooled sera from the Af + AIR groups and both were positive for Af-specific IgE by heterologous passive cutaneous anaphylaxis on rat skin. It is therefore, possible that mast cells degranulation was actively involved in the recruitment of eosinophils in the blood of Af + ETS group.

IL5, known to increase eosinophilic leukocytes (Galli *et al.*, 1991; Kurup *et al.*, 1994b; Hogaboam *et al.*, 1999) was detected from anti-CD3 stimulated cells. However, additional sources of IL5 from smoke stimulated mast cells were not measured *in vitro*, which may explain the lack of enhancement of IL5, after *in vitro* stimulation of lung cells from the Af + ETS group when compared to the Af + AIR group. There are other probable reasons for the higher levels of eosinophils in ETS exposed mice such as the production of the chemokines C10 and eotaxin. These mediators of eosinophilia were not measured in this study but were previously demonstrated in a murine model of ABPA (Hogaboam *et al.*, 1999). Proust *et al.* showed that intranasal anti-IL-5 treated OVA allergic mice had decreased BAL eosinophilia, but unchanged bronchial hyperresponsiveness and lung eosinophil sequestration (Shen *et al.*, 2003). This is in agreement with the lack of correlation of T cell-derived IL-5 with lung and peripheral eosinophilia and increased bronchial hyperresponsiveness seen in the present study.

Exposure of naïve mice to ETS alone produced an increase in airway responsiveness to acetylcholine confirming similar studies in humans showing that exposure to ETS alone causes nonspecific increases in airway reactivity to methacholine (Menon *et al.*, 1992). However, airway hyperresponsiveness was greater in the Af exposed groups. When we exposed naive mice to Af in the presence or absence of ETS, we observed the characteristic Th2 cytokine response and no IFN-γ after *in vitro* restimulation of homogenized lung with anti-CD3. Though the cytokine response was similar in both Af sensitized groups, there was a significant difference in airway hyperresponsiveness between them. Thus some synergism between Af and ETS in the establishment of airway hyperresponsiveness is suggested. In addition, these findings suggest that Af-induced increase in airway responsiveness represented an extremely sensitive endpoint that does not necessarily correlate with the cytokines observed from our *in vitro* stimulation of lung cells. Thus we believe cytokines not tested may have been responsible for the synergistic effect of the Af and ETS with respect to bronchial hyperresponsiveness. For example, the cytokine, IL-13 that was not examined in this study, instead of IL-4, was shown by others to be the most important cytokine in inducing bronchial hyperresponsiveness (Grunig *et al.*, 1998b). Other investigators have also demonstrated the importance of eosinophil in an allergic asthma model using IL-4 knock out mice in which neither IgE nor IL-4 were present, but airway hyperreactivity was demonstrated (Hogan *et al.*, 1997).

These data suggest that the profound airway hyperresponsiveness and eosinophilia observed in Af allergic mice is exacerbated by the ETS environment. The immunomodulatory effects of ETS and the relationship between allergen dosage and this effect pose interesting problems for future study.

### *Acknowledgements*

This research was supported in part by the University of California, Tobacco-Related Disease Research Program. The DNAX Research Institute is supported by the Schering Plough Corporation.

The authors thank Michael Goldsmith and Steve Teague for their technical assistance with the cigarette smoke generator system and the excellent care of the animals while they were housed in the exposure chambers. Animals were cared for under NIH guidelines. Animal care protocol was approved by the University of California, Davis Committee on Animal Use and Care.

We acknowledge Mario Alfaro for excellent technical assistance and Drs. Patricia Gilford and Gabriele Grunig for expert advice.

### *References*

Abrams, J. (1995) “Immunoenzymetric assay of mouse and human cytokines using NIP-labelled anti-cytokine antibodies”, In: Roico, R., ed, Current Protocols in Immunology (John Wiley and Sons, New York) Vol. 6.20.1.

Amdur, M.O. and Mead, J. (1958) “Mechanics of respiration in unanesthetized guinea pigs”, *Am. J. Physiol.* **192**, 364–368.

Braga, F. and Mota, I. (1976) “Homologous passive cutaneous anaphylaxis (PCA) in mice and heterologous PCA induced in rats with mouse IgE”, *Immunology* **30**, 655–669.

Coffman, R.L., Seymour, B.W.P., Hudak, S., Jackson, J. and Rennick, D. (1989) "Antibody to interleukin-5 inhibits helminth-induced eosinophilia in mice", *Science* **245**, 308–310.

Colley, D.G. (1972) "Schistosoma mansoni: eosinophilia and the development of lymphocyte blastogenesis in response to soluble egg antigen in inbred mice", *Exp. Parasitol.* **32**, 520–526.

El-Nawawy, A., Soliman, A.T., El-Azzouni, O., Amer, E., Demian, S. and El-Sayed, M. (1996) "Effect of passive smoking on frequency of respiratory illnesses and serum immunoglobulin-E (IgE) and interleuken-4 (IL-4) concentrations in exposed children", *J. Trop. Pediatr.* **42**, 166–168.

Galli, S.J., Gordon, J.R. and Wershil, B.K. (1991) "Cytokine production by mast cells and basophils", *Curr. Opin. Immunol.* **3**, 865–872.

Geng, Y., Savage, S.M., Johnson, L.J., Seagrave, J. and Sopori, M.L. (1995) "Effects of nicotine on the immune response. I. Chronic exposure to nicotine impairs antigen receptor-mediated signal transduction in lymphocytes", *Toxicol. Appl. Pharmacol.* **135**, 268–278.

Geng, Y., Savage, S.M., Razani-Boroujerdi, S. and Sopori, M.L. (1996) "Effects of nicotine on the immune response II. Chronic nicotine treatment induces T cell anergy", *J. Immunol.* **156**, 2384–2390.

Gilliland, F.D., Li, Y.F. and Peters, J.M. (2001) "Effects of maternal smoking during pregnancy and environmental tobacco smoke on asthma and wheezing in children", *Am. J. Respir. Crit. Care Med.* **163**, 429–436.

Grunig, G., Corry, D.B., Leach, M.W., Seymour, B.W.P., Kurup, V.P. and Rennick, D.M. (1997) "Interleukin-10 is a natural suppressor of cytokine production and inflammation in a murine model of allergic bronchopulmonary aspergillosis", *J. Exp. Med.* **185**, 1089–1099.

Grunig, G., Corry, D.B., Coffman, R.L., Rennick, D.M. and Kurup, V.P. (1998a) "Animal models of allergic bronchopulmonary aspergillosis", *Immun. Allergy Clin. N. Am.* **18**, 661–679.

Grunig, G., Warnock, M., Wakil, A.E., *et al.* (1998b) "Requirement for IL-13 independently of IL-4 in experimental asthma", *Science* **282**, 2261–2263.

Hogaboam, C.M., Gallinat, C.S., Taub, D.D., Strieter, R.M., Kunkel, S.L. and Lukacs, N.W. (1999) "Immunomodulatory role of C10 chemokine in a murine model of allergic bronchopulmonary aspergillosis", *J. Immunol.* **162**, 6071–6079.

Hogan, S.P., Mould, A., Kikutani, H., Ramsay, A.J. and Foster, P.S. (1997) "Aeroallergen-induced eosinophilic inflammation, lung damage, and airways hyperreactivity in mice can occur independently of IL-4 and allergen-specific immunoglobulins", *J. Clin. Investig.* **99**, 1329–1339.

Holt, P.G. (1987) "Immune and inflammatory function in cigarette smokers", *Thorax* **42**, 241–249.

Kauffman, H.F., Tomee, J.F.C., van der Werf, T.S., de Monchy, J.G.R. and Koeter, G.K. (1995) "Review of fungus-induced asthmatic reactions", *Am. J. Respir. Crit. Care Med.* **151**, 2109–2116.

Kurup, V.P., Mauze, S., Choi, H., Seymour, B.W.P. and Coffman, R.L. (1992) "A murine model of allergic bronchopulmonary aspergillosis with elevated eosinophils and IgE", *J. Immunol.* **148**, 3783–3788.

Kurup, V.P., Seymour, B.W.P., Choi, H. and Coffman, R.L. (1994a) "Particulate aspergillus fumigatus antigens elicit a Th2 response in BALB/c mice", *J. Allergy Clin. Immunol.* **93**, 1013–1020.

Kurup, V.P., Choi, H., Murali, P.S. and Coffman, R.L. (1994b) "IgE and eosinophil regulation in a murine model of allergic aspergillosis", *J. Leukoc. Biol.* **56**, 593–598.

Laufer, P., Fink, J.N., Bruns, W.T., *et al.* (1984) "Allergic bronchopulmonary aspergillosis in cystic fibrosis", *J. Allergy Clin. Immunol.* **73**, 44–48.

Martinez, F.D., Antognoni, G., Macri, F., *et al.* (1988) "Parental smoking enhances bronchial responsiveness in nine-year-old children", *Am. Rev. Respir. Dis.* **138**, 518–523.

McMenamin, C., Pimm, C., McKersey, M. and Holt, P.G. (1994) "Regulation of IgE responses to inhaled antigen in mice by antigen-specific gamma delta T cells", *Science* **265**, 1869–1871.

Menon, P., Rando, R.J., Stankus, R.P., Salvaggio, J.E. and Lehrer, S.B. (1992) "Passive cigarette smoke-challenge studies: increase in bronchial hyperreactivity", *J. Allergy Clin. Immunol.* **89**, 560–566.

Oryszczyn, M.P., Annesi-Maesano, I., Charpin, D., Paty, E., Maccario, J. and Kauffmann, F. (2000) "Relationships of active and passive smoking to total IgE in adults of the epidemiological study of the genetics and environment of asthma, bronchial hyperresponsiveness, and atopy (EGEA)", *Am. J. Respir. Crit. Care Med.* **161**, 1241–1246.

Ronchetti, R., Macri, F., Ciofetta, G., *et al.* (1990) "Increased serum IgE and increased prevalence of eosinophilia in 9-year-old children of smoking parents", *J. Allergy Clin. Immunol.* **86**, 400–407.

Ronchetti, R., Bonci, E., Cutrera, R., *et al.* (1992) "Enhanced allergic sensitisation related to parental smoking", *Arch. Dis. Child.* **67**, 496–500.

Rumold, R., Jyrala, M. and Diaz-Sanchez, D. (2001) "Secondhand smoke induces allergic sensitization in mice", *J. Immunol.* **167**, 4765–4770.

Seymour, B.W.P., Pinkerton, K.E., Friebertshauser, K.E., Coffman, R.L. and Gershwin, L.J. (1997) "Second-Hand smoke is an adjuvant for T helper-2 responses in a murine model of allergy", *J. Immunol.* **159**, 6169–6175.

Seymour, B.W.P., Gershwin, L.J. and Coffman, R.L. (1998) "Aerosol-induced immunoglobulin (ig)-E unresponsiveness to ovalbumin does not require CD8+ or T cell receptor (TCR)-γ/δ T-cells or interferon (IFN)-γ in a murine model of allergen sensitization", *J. Exp. Med.* **187**, 721–731.

Seymour, B.W.P., Friebertshauser, K.E., Peake, J.L., Pinkerton, K.E., Coffman, R.L. and Gershwin, L.J. (2002) "Gender differences in the allergic response of mice neonatally exposed to environmental tobacco smoke", *Dev. Immunol.* **9**, 47–54.

Shen, H.H., Ochkur, S.I., McGarry, M.P., *et al.* (2003) "A causative relationship exists between eosinophils and the development of allergic pulmonary pathologies in the mouse", *J. Immunol.* **170**, 3296–3305.

Shephard, R.J. (1992) "Respiratory irritation from environmental tobacco smoke", *Arch. Environ. Health* **47**, 123–130.

Sopori, M.L., Savage, S.M., Christner, R.F., Geng, Y.M. and Donaldson, L.A. (1993) "Cigarette smoke and the immune response: mechanism of nicotine induced immunosuppression", *Adv. Biosci.* **86**, 663–673.

Stedman, R.L. (1968) "The chemical composition of tobacco and tobacco smoke", *Chem. Rev.* **68**, 153–207.

Stone, R. (1992) "Bad news on second-hand smoke [news]", *Science* **257**, 607.

Teague, S.V., Pinkerton, K.E., Goldsmith, M., *et al.* (1994) "A sidestream cigarette smoke generation and exposure system for environmental tobacco smoke studies", *Inhal. Toxicol.* **6**, 79–93.

Thomas, W.R., Holt, P.G. and Keast, D. (1975) "Humoral immune response of mice with long-term exposure to cigarette smoke", *Arch. Environ. Health* **30**, 78–80.

Wang, J.M., Denis, M., Fournier, M. and Laviolette, M. (1994) "Experimental allergic bronchopulmonary aspergillosis in the mouse: immunological and histological features", *Scand. J. Immunol.* **39**, 19–26.

Wardlaw, A.J., Dunnette, S., Gleich, G.J., Collins, J.V. and Kay, A.B. (1988) "Eosinophils and mast cells in bronchoalveolar lavage in subjects with mild asthma. Relationship to bronchial hyperreactivity", *Am. Rev. Respir. Dis.* **137**, 62–69.

Witschi, H., Joad, J.P. and Pinkerton, K.E. (1997) "The toxicology of environmental tobacco smoke", *Annu. Rev. Pharmacol. Toxicol.* **37**, 29–52.

